# Ultrasonography-Guided Injection for Quadriceps Fat Pad Edema: Preliminary Report of a Six-Month Clinical and Radiological Follow-Up

**DOI:** 10.5334/jbr-btr.1148

**Published:** 2016-09-21

**Authors:** Zeynep Maras Ozdemir, Ustun Aydingoz, Mehmet Fatih Korkmaz, Volga Bayrakcı Tunay, Fatma Bilge Ergen, Ozgur Atay, Ozlem Baysal

**Affiliations:** 1 Inonu University School of Medicine, TR; 2 Hacettepe University School of Medicine, TR; 3 Hacettepe University School of Health Sciences, TR

**Keywords:** Quadriceps fat pad edema, Sonography-guided injection, Follow-up

## Abstract

**Purpose:**

To investigate efficacy and safety of ultrasonography-guided local corticosteroid and anesthetic injection followed by physical therapy for the management of quadriceps fat pad (QFP) edema.

**Materials and Methods:**

We prospectively evaluated 1671 knee MRI examinations in 1542 patients for QFP edema with mass effect, which was present in 109 (6.5%) knees. Participants were assigned into injection and therapy groups (both received the same physical therapy program). Injection group was first treated with ultrasonography-guided QFP injection of 1 mL corticosteroid and 1 mL local anesthetic agent. Patients were evaluated at baseline and 1-, 2-, 6-month follow-up for pain using static and dynamic visual analogue scale (VAS), suprapatellar tenderness, and QFP edema on MRI.

**Results:**

Final sample size consisted of 19 knees (injection group, 10; therapy group, 9) in 17 patients. An overall improvement was detected in both groups between baseline and final assessments. The injection group fared better than the therapy group in static VAS scores (3.33 ± 1.70 versus 0.56 ± 1.33), while there was no such difference for dynamic VAS. Incidence of suprapatellar tenderness decreased in both groups, statistically significantly in the injection group (from 100% to 0%). Pain reduction was greater in the injection group at the first month (88.9% – 90% good response versus 50% – 66.7% good response, *static-dynamic VAS scoring, respectively*), whereas there was no such superiority at the sixth month. No severe adverse events were identified.

**Conclusion:**

Ultrasonography-guided local injection followed by physical therapy is safe in the management of QFP edema; however, it is not superior to stand-alone physical therapy program in the long term.

## Introduction

The quadriceps (suprapatellar) fat pad (QFP) is an extrasynovial structure bordered anteriorly by the quadriceps tendon and posteriorly by the suprapatellar recess of the knee joint [1]. QFP edema characterized by diffuse enlargement on magnetic resonance imaging (MRI) may be analogous to Hoffa’s disease of the infrapatellar fat pad [2]. This inflammatory condition can cause anterior knee pain, although the relationship between edema and pain remains poorly understood [2,3].

Several studies have explored the frequency and MRI characteristics of QFP edema and/or the mass effect, and their relationships to knee pain and structural abnormalities of the knee [2,3,4]. Quadriceps fat pad (QFP) edema and the mass effect were not uncommon on MRI; however, any relationship between these conditions and anterior knee pain remains controversial [2,3,4]. Furthermore, Wang et al. recently suggested that alterations in the QFP mass effect and/or signal intensity in older patients may be a component of the pathological process of knee osteoarthritis [5]. However, although the MRI characteristics of quadriceps fat pad edema and the mass effect have been comprehensively described [2,3,4,5], only a few case reports on its management have appeared [2,3,4,6,7].

The aim of the present study was to explore the efficacy and safety of ultrasonography (US)-guided local corticosteroid and anesthetic injection, followed by physical therapy, to manage QFP edema. We present the preliminary report of the 6-month follow-up data.

## Materials and Methods

### Study design and participants

This prospective, consecutive-enrollment, non-randomized, two-center (one-center participated in an initial pilot study), controlled clinical study was performed with approval of our institutional ethical review board, and written informed consent was obtained from all patients from both centers. Two patients with anterior knee pain and QFP edema with mass effect on MRI (who also met the other eligibility criteria given below) first underwent US-guided QFP injection as described below in the first center.

**Figure 1:**
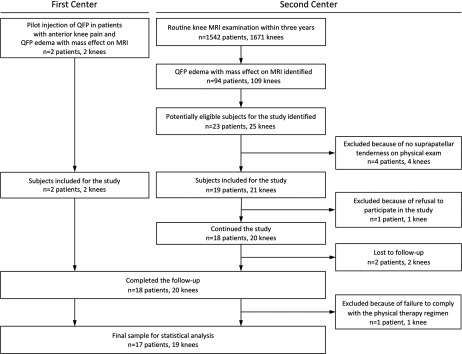
Flow diagram of the study.

We then prospectively evaluated in the second center 1,671 consecutive knee MRI examinations of 1,542 patients with various knee symptoms imaged between December 2011 and September 2014. A total of 109 knees (6.5%) of 94 patients (6.0%) exhibited QFP edema with a mass effect. A total number of 23 potentially eligible patients (24.5% of those with QFP edema displaying mass effect on MRI) with anterior knee pain were examined by a single orthopedic surgeon with four years of experience. A total number of 19 patients (20.2%) who met the clinical and radiological criteria were recruited for the study. However, one patient refused to participate, and the study commenced with the remaining 20 patients (22 knees; two from the first center, 20 from the second center). A total number of 18 patients (20 knees) were available for the 6-month follow-up; two patients (two knees, both from the second center) were lost to follow-up (Figure 1).

The inclusion criteria were QFP edema with a mass effect evident on MRI (see MRI examinations, below), the presence of anterior knee pain, suprapatellar tenderness (upon physical examination), and an age of 18 years or over. The exclusion criteria included pregnancy or lactation, a history of knee injury in the prior year, open or arthroscopic knee surgery in the prior year, any previous diagnostic or therapeutic intra-articular injection, a congenital or developmental disorder that could have disrupted knee shape or alignment, regular use of oral analgesics in the past 10 days and regular physical therapy in the three months prior to the current knee MRI. Other exclusion criteria were extensive lymphedema around the knee, a partial or full-thickness anterior or posterior cruciate ligament tear, a full-thickness collateral ligament or retinaculum tear, any displaced tear of the menisci or any loose intra-articular body.

### MRI examinations

All knee MRI examinations were performed on 1.5 T scanners (Magnetom Avanto or Symphony, Siemens Healthcare, Erlangen, Germany; Achieva, Philips, Best, the Netherlands) using dedicated knee coils. Each patient was placed supine on the MRI examination table with the knee at 10˚–15˚ of flexion. Our standard knee MRI protocol consisted of the following sequences: sagittal fat-suppressed (FS) proton density-weighted (PDW) fast spin echo (FSE), FS T2-weighted (T2W) FSE, and T1W FSE; coronal FS T2W FSE; and axial FS PDW FSE. On follow-up visits (see below), we ran only three sequences of the standard knee MRI protocol: sagittal FS PDW, FS T2W, and T1W.

**Figure 2:**
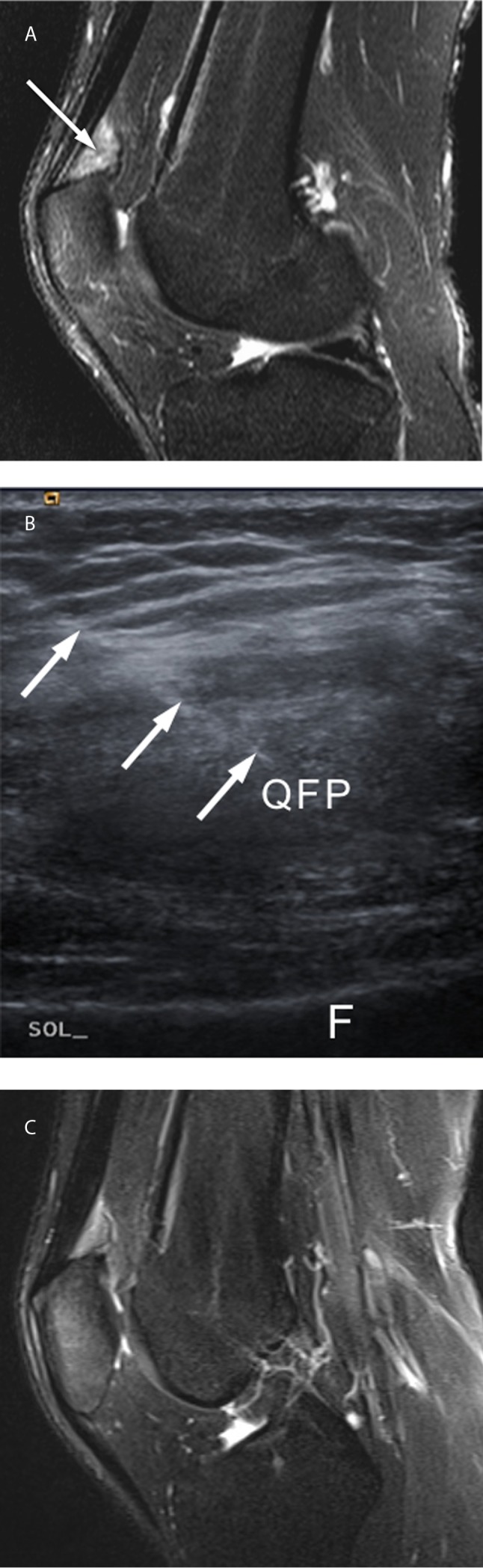
A 31-year-old male with anterior knee pain and suprapatellar tenderness on physical examination. Midsagittal FS T2W **(a)** MR image (TR/TE, 2728/60 ms) at baseline (Day 0) shows edema-like signal changes with mass effect (posterior bulging) at the quadriceps fat pad *(arrow)*. Oblique parasagittal US image **(b)** shows the injection needle *(arrow)* traversing through the quadriceps fat pat (QFP) (F, femur). Midsagittal FS T2W **(c)** MR image (TR/TE, 4020/64 ms) at 6-month follow-up (Day 180) shows persistence of edema-like signal changes at the quadriceps fat pad.

MRI examinations of 18 patients were reviewed by the same musculoskeletal radiologist (blinded) with two years of experience in musculoskeletal imaging. MRI of the remaining two patients have been evaluated by a senior musculoskeletal radiologist (blinded) with 17 years of experience in musculoskeletal MR imaging at the first center in which the pilot study of the QFP injection was conducted and physical therapy regime was designed. (These two patients, who also met the eligibility criteria, were included in the local injection group; however, they were not taken into account for the prevalence of QFP edema in the review of knee MRI examinations at the second center.) We considered by visual estimation that QFP edema was present when the signal intensity of at least two-thirds of the fat pad was greater than that of the surrounding normal muscle tissues on sagittal FS PDW or FS T2W images along with a mass effect – that was evident with a convex posterior fat-pad border on sagittal MR images (Figure 2a).

### Study groups

Patients were divided into two groups by reference to their treatment preferences. The therapy group included those who refused local injection, and the local injection group those who consented to injections of local anesthetic and steroid into the fat pad.

### Clinical evaluation of patients and treatment

The same orthopedic surgeon examined 18 participants within five days following routine knee MRI to determine whether point tenderness was evident over the suprapatellar region (0, no tenderness on palpation; 1, tenderness on palpation). The remaining two patients were examined similarly by a senior orthopedic surgeon (with 18 years of experience) at the pilot center.

All study participants attended a baseline session (Day 0) which included the following: collection of demographic information (age and gender); routine knee MRI; and scoring of pain on a 10-point visual analog scale (VAS) (0, no pain; 10, the worst pain imaginable) in a neutral (supine with the knee at 0° flexion) position (static VAS), and when standing up after having been in a seated or squatting position (dynamic VAS). Ultrasonography-guided injections (Figure 2b) were performed on 9 patients (9 knees) by the same radiologist, who had two years of experience in musculoskeletal imaging (blinded). At the pilot study center, a senior interventional radiologist had injected the remaining two patients. All examinations employed commercial ultrasound systems (Acuson Antares, Siemens Healthcare; Xario, Toshiba Medical Systems, Tochigi, Japan) fitted with high-frequency (5–13 MHz) linear-array transducers; the settings were adjusted to account for each patient’s subcutaneous tissue thickness.

A total of 2 mL [1 mL bupivacaine hydrochloride (Marcaine 0.5%, 5 mg/mL, blinded) and 1 mL methylprednisolone (40 mg/mL, blinded)] was injected into the fat pad of each consenting patient within five days of routine knee MRI after their pain scoring at the baseline session. All injections were administered with the patient supine and the knee under slight flexion (a small pillow was placed under the joint). Following administration of 3–5 mL prilocaine hydrochloride (Priloc 2%, 20 mg/mL, blinded) to establish local anesthesia, we advanced a 22-gauge needle obliquely, using an anteromedial approach, under ultrasonography control as described by Van Le and Harish [7]. We recorded any adverse event during or immediately after this process. Both groups underwent a physical therapy program initiated 15 days after baseline routine knee MRI; the program featured strengthening, stretching, and retraining.

At 1-month follow-up (Day 30), the following procedures were performed: limited knee MRI (see the three sequences below); physical examination of the knee, including assessment of suprapatellar tenderness, by the same respective orthopedic surgeon who had performed the initial physical examination at study centers; static and dynamic VAS scoring; and recording of adverse events. The physical therapy program continued. The same procedures were repeated at the 2-month (Day 60) and 6-month follow-up (Day 180) (Figure 2c).

### Efficacy and safety of treatment

The primary efficacy end-point was clinical improvement, as evaluated by changes in VAS scores from baseline to any follow-up visit after injection. The secondary efficacy parameters included between-group and intra-group variations in VAS scores, suprapatellar tenderness, and the extent of QFP edema with a mass effect evident on MRI at follow-up visits. In addition, we evaluated pain reduction by comparing differences between baseline and follow-up VAS scores. The responses to treatment were classified using the scheme of a recent retrospective study assessing the effectiveness of US-guided corticosteroid injection into the quadratus femoris muscle to treat patients with ischiofemoral impingement [8]: good response (VAS score reduction in pain level >2 with respect to the baseline), mild or partial improvement (VAS score reductions of 1 or 2 with respect to the baseline), or no improvement (with respect to the baseline). The safety parameters were the incidence, severity and outcomes of all adverse events.

### Statistical analysis

Data analysis was performed using SPSS for Windows, version 11.5 (SPSS Inc., Chicago, Illinois, USA). Data are shown as means ± standard deviations (SDs), or as numbers of cases with percentages, as appropriate. Student’s *t*-test was used to compare differences in age and anthropometric measures between the groups, and the Mann-Whitney U test was employed to compare VAS scores. Nominal data were analyzed using Fisher’s exact test. We employed the McNemar or Wilcoxon signed rank test, as appropriate, to determine whether differences evident between any two follow-up visits were significant. A *p* value less than 0.05 was considered to reflect statistical significance. The Bonferroni correction was applied to minimize Type 1 error upon all multiple comparisons (corrected *p* value, 0.05 divided by number of comparisons).

## Results

### Patients

We recruited 10 (55.5%) males and 8 (44.4%) females (a total of 20 knees; bilateral knees in two females). Follow-up data were collected from all 18, but we excluded one male patient from analysis when he disclosed non-compliance with the physical therapy program (Figure 1). Thus, the final sample was composed of 19 knees (17 patients), of which 10 had been injected (10 patients, mean [SD] age, 44.0 [9.1] years; range, 29–54 years). Nine knees were included in the therapy group (7 patients, mean [SD] age, 34.1 [8.2] years; range, 24–50 years).

The mean age and body mass index (BMI) were significantly higher in the injected than in the therapy group, but neither gender nor knee sidedness differed. The demographic data are summarized in Table 1.

**Table 1:** Summary of demographic data.

	Injection group (n = 10 knees in 10 patients)	Therapy group (n = 9 knees in 7 patients)	*p* value

Age (years)	44.0 ± 9.1	34.1 ± 8.2	0.024^a^
Gender			0.070^b^
Male	7 (70.0%)	2 (22.2%)	
Female	3 (30.0%)	7 (77.8%)	
Height (cm)	172.0 ± 8.5	164.8 ± 9.1	0.092^a^
Weight (kg)	82.0 ± 14.2	66.0 ± 11.2	0.015^a^
Body mass index (BMI) (kg/m^2^)	27.7 ± 4.1	24.1 ± 1.9	0.029^a^

^a^ Student’s t test. ^b^ Fisher’s exact test.

### Baseline and follow-up data

#### Pain

**Figure 3:**
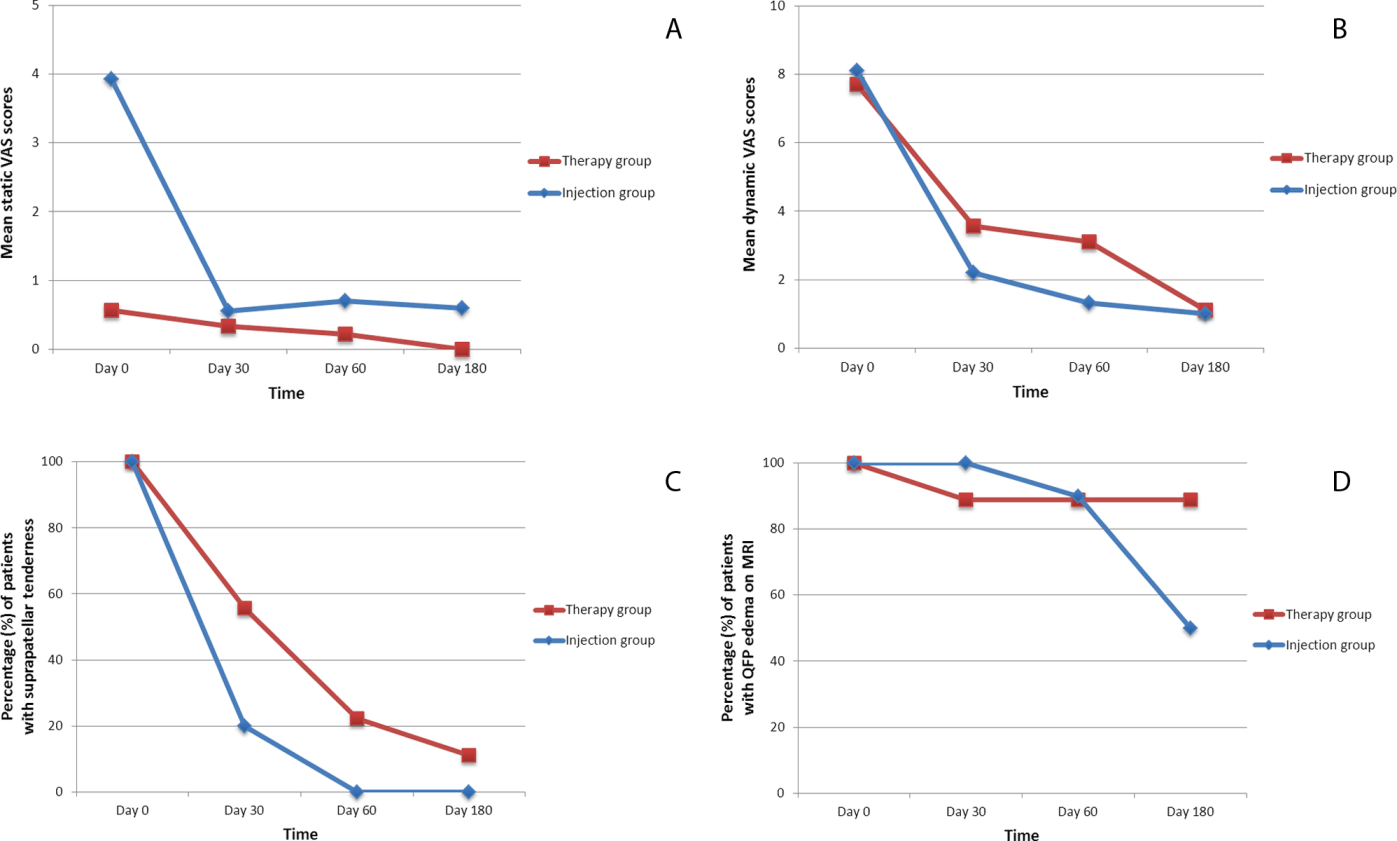
Graphs of timeline of clinical and radiological findings in study groups. Changes from baseline **(a, b)** in static and dynamic VAS scores. Changes from baseline **(c)** in presence of suprapatellar tenderness. Changes from baseline **(d)** in quadriceps fat pad (QFP) edema on MRI. Note that x-axes are not to scale; and y-axes on **a** and **b** represent mean VAS scores, while they denote percentage of patients with the presence of a condition on **c** and **d**.

At baseline, the mean static VAS score of the injected group was significantly higher than that of the therapy group (*p* = 0.003) (Table 2). The mean static VAS score was reduced (compared to baseline) at the time of final assessment (6 months) in both groups, despite the fact that the mean differences in static VAS scores did not differ significantly between consecutive follow-ups in either group (*p* > 0.0041, after Bonferroni correction) (Figure 3a). Moreover, when compared to the therapy group, the injected group showed significant decreases in mean static VAS scores at the 1-, 2-, and 6-month follow-up visits against baseline (*p* < 0.001, *p* < 0.001, and *p* = 0.004) (Figure 3a).

**Table 2:** VAS scores of patients at baseline and during follow-up.

	Injection group	Therapy group	*p* value^b^

**Static VAS scores^a^**			
Day 0 (baseline)	3.93 ± 2.77	0.56 ± 1.33	0.003^b^
Day 30	0.55 ± 1.06	0.33 ± 0.71	0.780
Day 60	0.70 ± 0.95	0.22 ± 0.67	0.211
Day 180	0.60 ± 1.07	0.00 ± 0.00	0.278
**Dynamic VAS scores^a^**			
Day 0 (baseline)	8.10 ± 1.52	7.71 ± 1.99	0.604
Day 30	2.20 ± 2.45	3.56 ± 1.51	0.035
Day 60	1.32 ± 1.54	3.11 ± 1.54	0.035
Day 180	1.00 ± 1.33	1.11 ± 1.27	0.780

VAS, visual analog scale. ^a^ Values given as mean ± standard deviation. ^b ^*p* values denote comparison of groups at given timepoints; statistically significant value is *p* < 0.0125 according to Mann Whitney U test and Bonferroni correction.

The dynamic VAS scores were similar in both groups at baseline (*p* > 0.0125, after Bonferroni correction) (Table 2). The mean dynamic VAS scores of both groups decreased at the time of the final assessment (6 months), compared to baseline values (Figure 3b). However, no significant within-group difference was evident in either group between consecutive follow-ups. The dynamic VAS score of the therapy group significantly decreased, compared to that of the injected group, specifically between the 2- and 6-month follow-up visits (*p* = 0.004).

No significant between-group difference was evident in pain reduction at the 1-, 2-, and 6-month follow-ups, compared to the baseline values. However, in the injected group, the static VAS scores improved at the 1- and 2-month follow-ups to a greater extent than in the therapy group, but this was not the case at the 6-month follow-up (88.9% of patients in the injected group gave *good* scores at the 1- and 2-month follow-ups, and 77.8% at the 6-month follow-up; the respective figures for the therapy group were 50% and 100%). In terms of changes in dynamic VAS scores, the injected group reported more relief at the 1-month follow-up, but the scores of both groups were the same at the 2- and 6-month follow-ups (at 1 month, 90% of injected patients gave *good* scores, compared to 66.7% of therapy patients; at the 2- and 6-month follow-ups, all patients in both groups gave *good* scores) (Table 3).

**Table 3:** Response to treatment as derived from changes in static and dynamic VAS scoring^a^ during follow-up.

	Injection group^b^				Therapy group^b^			
	Good response	Mild improvement	No improvement		Good response	Mild improvement	No improvement	*p* value^c^

**Static VAS scoring**								
Day 30	88.9	11.1	–		50.0	50.0	–	0.436
Day 60	88.9	11.1	–		50.0	50.0	–	0.436
Day 180	77.8	22.2	–		100.0	–	–	0.727
**Dynamic VAS scoring**								
Day 30	90.0	10.0	–		66.7	11.1	22.2	0.356
Day 60	100.0	–	–		100.0	–	–	–
Day 180	100.0	–	–		100.0	–	–	–

VAS, visual analog scale. ^a^ Response to treatment determined according to the scheme used at reference 8: good response (reduction in pain level >2 VAS scores with respect to the baseline; mild improvement (reductions of 1 or 2 VAS scores with respect to the baseline), or no improvement (with respect to the baseline). ^b^ Percentages of patients. ^c^ Pertaining to differences between study groups; statistically significant value is *p* < 0.0083 according to Bonferroni correction.

#### Suprapatellar tenderness

This incidence decreased in both groups, particularly the injected group, at the time of the final (6-month) assessment, compared to the baseline values (Figure 3c). The decrease was significant in the injected group at both the 2- and 6-month follow-up visits, compared to baseline (*p* = 0.002 and *p* = 0.002, respectively), but no significant between-visit difference was evident in the therapy group.

#### Quadriceps fat pad edema on MRI

The incidence of QFP edema on MRI did not significantly differ between or within the two groups throughout the study period (Figure 3d). The incidence decreased, compared to baseline, in both groups, but more prominently in the injected group at the time of final assessment (6-month) (Figure 3d). Edema decreased (i.e., no longer fulfilled the initial MRI criterion) in four of eight of injected and one of nine of therapy patients by 6 months.

#### Safety parameters

The treatments were tolerated well and no severe adverse event was noted; however, almost all patients (9/10, 90%) in the injected group reported markedly increased local pain (i.e., more severe than at baseline) several hours after intra-fat pad injection that disappeared within 1–2 days.

## Discussion

In this prospective, controlled preliminary clinical study to assess the efficacy and safety of US-guided intra-fat pad injection in symptomatic patients with QFP edema, we found clinical improvements in both the injection and therapy groups after six months of treatment. Pain reduction was greater in the injected group at the 1-month, but not at the 6-month, follow-up. Thus, US-guided local injection followed by physical therapy is an effective and reliable treatment for QFP edema, and injection affords more effective short-term pain reduction. However, local injection is no better than stand-alone physical therapy in the longer term.

Few data on the management of patients with QFP edema and a mass effect are available. Previous articles discussed the MRI characteristics of such patients and relationships thereof with particular structural abnormalities of the knee [2,3,4,5]. Roth et al. prescribed physical therapy for all patients exhibiting QFP enlargement with anterior knee pain [3]. Local intra-articular corticosteroid injection (one patient) and the use of an unknown medication by another patient mildly improved the symptoms [3]. Tsavalas and Karantanas reported complete resolution of anterior knee pain in one patient after a single US-guided intra-fat pad injection of corticosteroid [4]. In addition, Sirvanci and Ganiyusufoglu reported that CT-guided *steroid* injections into four patients eliminated all symptoms, with no recurrence; however, the details of the injections and follow-up duration were not given [6]. Additionally, complete resolution of symptoms was achieved upon surgical resection of the fat pad in a patient with histological findings similar to those of Hoffa’s disease [2].

To date, no report has detailed the nature and amount of medication given, the injection method, or clinical follow-up data on QFP edema, with the exception of one case report [7]. We studied 19 knees of 17 patients and explored the utility of local drug therapy. Although the VAS scores did not differ significantly between the two groups, both groups showed clinical and radiological improvements compared to baseline. The improvements were more marked in the injected group, and significant reductions in static VAS scores were evident at months 1, 2, and 6, compared to baseline. However, this may simply reflect the higher mean static VAS score of the injected group at baseline. Upon subanalysis of pain reduction, local drug therapy was more successful in the early post-injection period (1 month); however, no difference was evident at later periods. Moreover, the therapy group achieved greater static pain reduction at 6 months; the dynamic VAS scores of both groups were similar at this time. We speculate that this may be attributable to the possibility of a lower-than-full compliance with the physical therapy regimen by injected patients after rapid relief was afforded by QFP injection (patients were taken at their word during the follow-up visits as to their interval compliance with the physical therapy regimen; in fact, one patient was excluded from the study upon his disclosure of non-compliance).

Although injections afforded rapid relief, consistent with the established efficacy of corticosteroids for reducing inflammation and relieving pain in the early stages of a wide array of inflammatory conditions, physical therapy alone in our study was associated with gradual clinical improvement over time. Restoration of patellar tracking biomechanics by active (quadriceps retraining) and passive (strengthening, stretching) exercises improves lower limb control and patellar congruence of the joint, contributing to pain improvement [9]. Thus, local injection followed by physical therapy is both effective and reliable for treating anterior knee pain associated with QFP edema, particularly in patients requiring rapid pain relief.

MRI is the most useful tool for diagnosis of soft tissue abnormalities such as QFP edema with a mass effect. Several previous studies have yielded consistent estimates of the prevalence of the condition where only QFP mass effect, but not edema-like signal, was assessed. This was 12% (11/92 knee MRI examinations) in the work of Roth et al. [3]; 13.8% (110/685 patients) in the study of Tsavalas and Karantanas [4]; and 13% (29/736 older patients) in the work of Wang et al. [5]. However, we diagnosed the condition as described by Shabshin et al. (i.e., taking into account not only mass effect but also frank edema-like signal on MRI) and found a prevalence of 6.5% (109/1,671 MRI examinations), closer to the 4.2% (32/770 MRI examinations) reported by Shabshin et al. [2]. Except for a single case in a study report [2], there is no mention on the temporal course of QFP edema (with or without treatment) on imaging in the literature. It is interesting to note that despite treatment QFP edema on MRI persisted (fulfilling our MRI criteria) in eight of nine knees in the therapy group and four of eight knees in the injection group. Even in the remaining knees in both groups some QFP edema persisted on MRI although no longer fulfilling our MRI criteria of QFP edema for initial eligibility for inclusion in the study.

Any clinical relationship between QFP edema and anterior knee pain remains controversial. Roth et al. [3] reported that the QFP mass effect was in fact associated with anterior knee pain (45.4%, 5/11 knee MRI examinations); Shabshin et al. [2] noted that QFP edema with the mass effect might cause anterior knee pain (27.6%, 8/29 patients); however, Tsavalas and Karantanas [4] suggested that the condition was rarely associated with anterior knee pain (5.4%, 6/110 patients). A comprehensive prospective study (904 patients) on associations between the MRI abnormalities of QFP edema and knee symptoms and structures, in older subjects, revealed that QFP abnormalities were positively associated with knee pain, particularly in those with radiographic osteoarthritis [5]. The last cited authors also found that QFP edema with a mass effect was associated with radiographic osteoarthritis, and suggested that QFP abnormalities could be components of the late-stage pathology of knee osteoarthritis [5]. It was beyond the capacity of the present study to explore relationships between QFP abnormalities and anterior knee pain, patellofemoral cartilage abnormalities, or joint osteoarthritis (our final study group included in the second center, which had the majority of cases, only about 15.6% of cases with QFP edema and mass effect on MRI). Only two knees (in two patients) in our study groups exhibited some patellofemoral compartment cartilage degeneration (and they were restricted to the patellar side); remaining knees (89% of all) were free of patellofemoral compartment cartilage degeneration. Our results indicate that QFP edema with a mass effect may indeed be associated with anterior knee pain and suprapatellar tenderness, with rapid clinical improvement following direct injection of the QFP in our study lending extra credence to such an association.

Although the causes of QFP abnormalities remain poorly understood, several theories have been advanced. Anatomical discordance of the extensor mechanism (and/or a possibly abnormal extensor mechanism) has been suggested to trigger OFP enlargement, particularly in subjects with bilateral clinical and radiological findings [3]. However, to date, no relationships among the presence of QFP edema with a mass effect, anatomical measures of the extensor mechanism, or patellofemoral malalignment have been noted [2,3]. Another theory is that QFP enlargement is triggered by intra-articular changes caused by co-existing knee abnormalities, such as chondromalacia, synovitis, or osteoarthritis. Although three preliminary studies found no significant associations between patellofemoral cartilage abnormalities and patellofemoral joint osteoarthritis [2,3,4], one recent study reported that QFP edema with a mass effect was significantly associated with radiographic osteoarthritis and narrowing of the joint space of the medial tibiofemoral compartment [5]. Authors of the last cited study suggested that QFP abnormalities might be late-stage components of knee osteoarthritis [5]. Acute and chronic repetitive trauma, or overuse, may also cause QFP abnormalities [3,4], as is the case in Hoffa’s disease [10]. The use of high knee flexion angles and an unusual sitting position assumed during daily life can create contact between the proximal patellar pole and the femur [11], and articulate the QFP with the trochlea [1]. This has been considered to be a risk factor for QFP edema and inflammation; many patients in our study reported extensive knee flexion (prostration during prayer, or sitting cross-legged or on bent knees) in their daily routine. Therefore, we agree with Roth et al. [3], who suggested that excessive high-angle knee flexion can trigger QFP edema and inflammation because of repetitive microtrauma or development of an overuse injury, causing mechanical impingement of bone onto soft tissue.

Our study has several limitations. First, the statistical power is low because of our relatively small sample size. Our ability to detect significant changes was thus reduced. Second, non-randomized design of the study caused patient selection bias. Third, the between-group differences in BMI and age might have increased the error variance and reduced the ability to detect small differences. Fourth, the higher baseline static VAS scores of the injected group and the possibility of lower-than-full-compliance with the physical therapy regimen (particularly by the injected group, after rapid relief) might have somewhat compromised our ability to detect the true effects of treatment, and differences between the study groups. We did, however, specifically question at follow up visits the patients’ compliance with the physical therapy regimen and excluded from the study one patient who disclosed none compliance. Fifth, we did not attempt to exclude patients with patellofemoral compartment cartilage degeneration, which may be encountered in persons at the age groups as in our study and might have been the cause of (or at least have contributed to) anterior knee pain; this condition (seen in two of our cases) by itself might have benefited from our treatment protocol. Although the lack of a placebo control group may be considered as yet another limitation, placebo injections in humans are not acceptable according to our institutional review board’s code of conduct. Finally, we did not assess patients for longer than six months. Therefore, whether the effect of local drug injection extends beyond this time is unknown.

Despite these limitations, the results of our controlled preliminary clinical study show that US-guided local injection followed by physical therapy is effective and well-tolerated when used to manage QFP edema. Injection is effective in terms of pain reduction in the near-term. However, local injection is not superior to physical therapy alone in the longer term.

## References

[1] Staeubli HU, Bollmann C, Kreutz R, Becker W, Rauschning W (1999). Quantification of intact quadriceps tendon, insertion, and suprapatellar fat pad: MR arthrography, anatomy and cryosections in the sagittal plane. AJR.

[2] Shabshin N, Schweitzer ME, Morrison WB (2006). Quadriceps fat pad edema: significance on magnetic resonance images of the knee. Skeletal Radiol.

[3] Roth C, Jacobson J, Jamadar D, Caoili E, Morag Y, Housner J (2004). Quadriceps fat pad signal intensity and enlargement on MRI: prevalence and associated findings. AJR.

[4] Tsavalas N, Karantanas AH (2013). Suprapatellar fat-pad mass effect: MRI findings and correlation with anterior knee pain. AJR.

[5] Wang J, Han W, Wang X (2014). Mass effect and signal intensity alteration in the suprapatellar fat pad: associations with knee symptoms and structure. Osteoarthritis Cartilage.

[6] Sirvanci M, Ganiyusufoğlu AK (2005). Quadriceps fat pad signal intensity and enlargement on MRI. Letter to the editor. AJR.

[7] Van Le B, Harish S (2009). Quadriceps fat pad edema: sonographic depiction and sonographically guided steroid injection. J Ultrasound Med.

[8] Backer MW, Lee KS, Blankenbaker DG, Kijowski R, Keene JS (2014). Correlation of ultrasound-guided corticosteroid injection of the quadratus femoris with MRI findings of ischiofemoral impingement. AJR.

[9] Dragoo JL, Johnson C, McConnell J (2012). Evaluation and treatment of disorders of the infrapatellar fat pad. Sports Med.

[10] Jacobson JA, Lenchik L, Ruhoy MK, Schweitzer ME, Resnick D (1997). MR imaging of the infrapatellar fat pad of Hoffa. RadioGraphics.

[11] Huberti HH, Hayes WC, Stone JL, Shybut GT (1984). Force ratios in the quadriceps tendon and ligamentum patellae. J Orthop Res.

